# 

**Published:** 1905-08-05

**Authors:** 


### NOTICE TO CORRESPONDENTS.

All MSS., Letters, Books for Review, and other matters intended for the Editor should be addressed The Editor, "The Hospital," The Hospital Buildings, 28 & 29 Southampton Street, Strand, W.C.

The Editor cannot undertake to return rejected MSS., even when accompanied by stamped directed envelope.

**Notice to Advertisers and Subscribers.**

All Advertisements, Orders for Copies of The Hospital, and Business Communications should be addressed to **THE MANAGER (Not to the Editor),** The Hospital, 28 & 29 Southampton Street, Strand, London, W.C.

The Cost of Subscription, post free, is at follows:—Per week, 3½d.; per uarter, 3s. 3d.; per half-year, 6s. 6d.; per annum, 13s. Foreign and olonial:—Per annum, 19s. 6d., payable, in all cases, in advance.

**NOTICE.—Vol. XXXVII. of "The Hospital" October 1904 to March 1905), in handsome cloth binding, will shortly be on sale at the office of this Journal, price 10s. 6d. Cases for binding the Numbers contained in this Volume 2s. Index to Vol. XXXVII., 3½d., post free.**

**The Monthly Part for August is now ready. Price 1s., by post, 1s. 3d.**

### Medical Appointments.

**Alec H. Brewer**, M.R.C.S., L.R.C.P.Lond., Anæsthetist to the Samaritan Free Hospital for Women. **R. A. Earle**, L.S.A. Lond., Medical Officer and Public Vaccinator for the Weston-super-Mare District of the Axbridge Union. **Trevethan Frampton**, F.R.C.P., M.R.C.S., Clinical Assistant to the Chelsea Hospital for Women. **W. J. Hancock**, M.R.C.S., L.R.C.P.Lond., Certifying Surgeon under the Factory and Workshop Act for the Stalybridge District of the county of Chester. **R. A. L. Hill**, M.R.C.S.Eng., L.R.C.P.Lond., Certifying Surgeon under the Factory and Workshop Act for the Wimbledon District of the county of Surrey. **C. P. Lapage**, M.D.Vict., Medical Officer to the Out-patient Department of the Manchester Children's Hospital. **Lawrie H. McGavin**, F.R.C.S.Eng., Surgeon to the In-patients on the Staff of the *Dreadnought* Hospital, Greenwich. **Marcus S. Paterson**, M.B., B.S., M.R.C.S., L.R.C.P., Medical Superintendent of the Brompton Hospital Sanatorium, Frimley, Surrey. **G. M. G. Swainson**, F.R.C.S., Assistant Surgeon to the North-Eastern Hospital for Children, Hackney Road. **L. Kirkby Thomas**, M.R.C.S., L.R.C.P.Lond., Anæsthetist to the Birmingham and Midland Hospital for Women. **Frank Edward Tylecote**, M.D.Vict., Medical Registrar to the Manchester Royal Infirmary. **Arthur Westerman**, M.D.Aberd., Medical Officer to the City of London and East London Dispensary.

### NEW BOOKS FOR NURSES.

*Published by*
THE SCIENTIFIC PRESS, LTD.
28 & 29 Southampton Street,
Strand, London, W.C.

### SIMPLE SANITATION:
**The Practical Application of the Laws of Health to Small Dwellings.**

By M. LOANE.

With an Introductory Chapter on Sanitary Legislation by Dr. A. Mearns Fraser, Medical Officer of Health, Portsmouth. Limp cloth, 1/- net, 1/2 post free.

This work should prove of great practical assistance to District Nurses and Midwives, as it contains Chapters on Sanitary Legislation, Water, Air and Ventilation, Drainage and Disposal of Refuse, Household Management, Personal Hygiene, Disinfectants, &c.

### OUTLINES OF ROUTINE IN DISTRICT NURSING.

Drawn up for the use of District Probationers and Private Nurses.

By M. LOANE,
Superintendent of District Nurses, Portsmouth.
Second Edition, Rewritten and Enlarged.
Limp cloth 1/6 net, 1/9 post free.

This book contains outlines of the routine proved by long practical experience to be the most desirable, and will be very useful to all Nurses engaged in Private or District Nursing.

### HINTS TO NURSES ON TROPICAL FEVERS.

By S. F. PALLARD.
Cloth, 1/6 net, 1/8 post free.

This work should prove of the utmost value to Nurses and also to families in the Tropics, as the information it contains is so lucid and terse as to be readily understood.

### A PRACTICAL GUIDE TO COOKERY IN WEST AFRICA AND THE TROPICS.

By Sister COCKBURN,
Late Colonial Nursing Service, Sierra Leone, West Africa.
Cloth boards, 3/- net, 3/3 post free.

A handy practical book, easy of reference, the recipes being easily prepared and likely to be acceptable as providing a pleasant change in the rather limited menu of the ordinary West African cuisine, particularly in the case of invalids or convalescents.

### The Book for the C.M.B.

### A COMPLETE HANDBOOK OF MIDWIFERY.

By J. K. WATSON, M.D.,

Author of "A Handbook for Nurses."

143 Illustrations.

6/- net, 6/4 post free. 6/- net, 6/4 post free.

This book has been compiled in accordance with the requirements of the Central Midwives Board, and will prove not only the best text-book for those entering for the next exam., but is so complete as to be of greatest value to the Qualified Midwife for immediate reference in cases of emergency.

*The above works can be obtained of all Booksellers, or of*
**The SCIENTIFIC PRESS, Ltd., 28 and 29 Southampton St., Strand, London, W.C.**

### The NURSING MIRROR.

**The Nurse's Own Paper.**
**Price ONE PENNY Weekly.**

Of all Booksellers and Newsagents, or posted direct from the Office.

*Write for Subscription Rates to The Manager,*
**THE NURSING MIRROR, The Hospital Building, 28 & 29 Southampton Street, Strand, London, W.C.**

